# Genomic Signatures for Avian H7N9 Viruses Adapting to Humans

**DOI:** 10.1371/journal.pone.0148432

**Published:** 2016-02-04

**Authors:** Guang-Wu Chen, Shu-Ming Kuo, Shu-Li Yang, Yu-Nong Gong, Mei-Ren Hsiao, Yi-Chun Liu, Shin-Ru Shih, Kuo-Chien Tsao

**Affiliations:** 1 Department of Computer Science and Information Engineering, School of Electrical and Computer Engineering, College of Engineering, Chang Gung University, Taoyuan, Taiwan; 2 Research Center for Emerging Viral Infections, College of Medicine, Chang Gung University, Taoyuan, Taiwan; 3 Department of Laboratory Medicine, Linkou Chang Gung Memorial Hospital, Taoyuan, Taiwan; 4 Department of Medical Biotechnology and Laboratory Science, College of Medicine, Chang Gung University, Taoyuan, Taiwan; Centers for Disease Control and Prevention, UNITED STATES

## Abstract

An avian influenza A H7N9 virus emerged in March 2013 and caused a remarkable number of human fatalities. Genome variability in these viruses may provide insights into host adaptability. We scanned over 140 genomes of the H7N9 viruses isolated from humans and identified 104 positions that exhibited seven or more amino acid substitutions. Approximately half of these substitutions were identified in the influenza ribonucleoprotein (RNP) complex. Although PB2 627K of the avian virus promotes replication in humans, 45 of the 147 investigated PB2 sequences retained the E signature at this position, which is an avian characteristic. We discovered 10 PB2 substitutions that covaried with K627E. An RNP activity assay showed that Q591K, D701N, and M535L restored the polymerase activity in human cells when 627K transformed to an avian-like E. Genomic analysis of the human-isolated avian influenza virus is crucial in assessing genome variability, because relationships between position-specific variations can be observed and explored. In this study, we observed alternative positions that can potentially compensate for PB2 627K, a well-known marker for cross-species infection. An RNP assay suggested Q591K, D701N, and M535L as potential markers for an H7N9 virus capable of infecting humans.

## Introduction

A novel influenza A H7N9 virus emerged in Eastern China in March 2013, and 241 of the 676 laboratory-confirmed cases reported until July 2015 resulted in death, a remarkable 36% case fatality rate. Most cases were from China, except four from the Taipei Centers for Disease Control (Taipei CDC), twelve from the Centre for Health Protection, Hong Kong SAR, one Chinese traveler reported by Malaysia, and 2 Canadian travelers returning from China. The first Taiwanese H7N9 infection was reported in April 2013 in a businessman returning from the Jiangsu province of China [1, 2]. The second and third infections were reported in December 2013 and April 2014 in Chinese tourists [3, 4]. The fourth H7N9 infection was reported on April 25, 2014, in a businessman with a history of travel to China [5]. All four recovered after hospitalization with clinical treatments except that the 2^nd^ patient died of complication of septic shock from bacterial pneumonia.

Introduction of genetic variations in viral ribonucleoprotein (RNP) genes is a major virulence determinant for avian influenza viruses. Three genetic substitutions, PB2 E627K, D701N, and Q591R, have been reported to affect host cell tropism [6–10]. In particular, a single substitution of glutamic acid (E) to lysine (K) at PB2 627 of avian influenza viruses considerably enhances their polymerase activity, virus replication, transmission ability, and pathogenicity in mammalian cells and mice [11–17].

The novel influenza A H7N9 virus is an avian virus to affect humans [18, 19]. In mice experiments, the virus was found more pathogenic than an avian H7N9 virus (A/duck/Gunma/466/2011) and a representative H1N1 virus (A/California/4/2009) [20]. In ferret, A/Shanghai/2/2013 could replicate well in the upper and lower respiratory tracts to high titers for 6 to 7 days [21]. The virus was found directly transmitted by contact, and was less efficient in airborne [21, 22]. Regardless, one isolate from a patient in Anhui was shown highly transmissible between ferrets by respiratory droplets [23]. H7N9 virus was found able to infect but did not replicate well in pigs after intranasal inoculation [20, 21]. Neither was it able to further transmit to other pigs [21]. It was reported that a single Q226L mutation (H3 numbering) on the influenza A hemagglutinin (HA) enabled the H7N9 viruses to have a mixed α-2,3/α-2,6 receptor preference, which increased binding to mammalian-like receptors in the human upper airway [24]. Moreover, the replication-promoting PB2 E627K mutation dominated the H7N9 patient isolates [25, 26]. Other PB2 mutations including Q591K, and D701N were also investigated for their enhancing polymerase activity in human 293T cells [27].

In this study, we determine the genome of the fourth H7N9 isolate from Chang Gung Memorial Hospital (CGMH) and describe the genome diversity of these Taiwanese isolates. We subsequently assess the genetic diversity of H7N9 genomes from cases reported between March 2013 and April 2015. In our previous studies, we performed large-scale scanning of the influenza A virus genomes and summarized a list of species-associated signatures to distinguish human and avian viruses [18, 28, 29]. Using these signature positions, we investigated how most of the avian signatures were retained in the H7N9 viruses and which signature positions may become characteristic to humans. Moreover, we performed a genome-wide scan to summarize the genetic diversity among all H7N9 viral proteins and focused on PB2 mutations that potentially enable the virus to infect humans. Finally, a reporter assay was performed to assess the influence of mutations on RNP activities.

## Materials and Methods

### Ethics Statement

The present study aimed to characterize the genomic heterogeneity of human H7N9 viruses in the four Taiwanese patients, as well as all reported H7N9 genomes from the public databases since March 2013. Three genomes each belongs to the first three Taiwanese patients were among the database genomes we collected. These genomic sequence records are available to the general public through their web services (The Influenza Virus Resource, and the Global Initiative on Sharing Avian Influenza Data). No patient information were ever made available through these services. The two genomes sequenced in this study were derived from an H7N9 virus isolate deposited to a virus bank maintained in the Clinical Virology Laboratory of CGMH. Such isolates were cultured from the clinical specimens collected by physicians for the purpose of medical diagnosis or public health investigation. Clinical information from any of these specimens were anonymized and de-identified prior to their inclusion to the virus bank such that the authors had no access to any identifying information at any time.

### Virus Isolation

The virus was isolated from sputum samples of the aforementioned patient admitted to the CGMH by using the Madin-Darby canine kidney (MDCK) cell line maintained in a Dulbecco’s modified Eagle’s medium (DMEM, Gibco, Grand Island, NY, USA) containing 0.1 mg/mL trypsin. For virus propagation, 200-μL of the sputum samples were injected into the allantoic cavity of 10- to 11-day-old embryonated eggs and incubated at 37°C for 3 days; subsequently, the allantoic fluid of the inoculated chicken eggs was harvested. Labels 4-CGMH1 and 4-CGMH2 represent the viruses isolated from egg passage 1 and MDCK cell passage 2, respectively, and “4” represents the fourth case of H7N9 in Taiwan.

### RT-PCR and Sequencing

Viral RNAs were extracted from the cell culture supernatant or the allantoic fluid of the inoculated chicken eggs according to the manufacturer’s instructions using a QIAamp Viral RNA Mini Kit (Qiagen, Valencia, CA, USA). The RNA was reverse transcribed into a cDNA by using SuperScript III reverse transcriptase (RT) (Invitrogen, Carlsbad, CA, USA). The polymerase chain reaction (PCR) was performed using a proofreading DNA polymerase KOD-plus (Toyobo, Osaka, Japan) and the specific primers listed in S1 Table. The following PCR conditions were applied: 40 cycles of 94°C for 30 s, 50°C or 55°C for 30 s, and 68°C for 2 min (BioMetra Thermocycler, Biometra, Göttingen, Germany). The PCR products were isolated through electrophoresis on 1% agarose gel, and appropriate-size amplicons were excised from the gel and purified using a QIAquick gel extraction kit (Qiagen, Valencia, CA, USA). Nucleotide sequencing was performed according to the manufacturer’s protocols using the BigDye terminator cycle sequencing kit (Version 3.1, Applied Biosystems, Carlsbad, CA, USA). The nucleotide sequences were assembled using the SeqMan program (DNASTAR, Madison, WI, USA).

### GenBank Accession Numbers

The full genome sequences of A/Taiwan/4-CGMH1/2014 and A/Taiwan/4-CGMH2/2014 are available in GenBank (KM244508–KM244523). Other H7N9 sequences included in this study were downloaded from the Influenza Virus Resource of National Biotechnology Information Center (NCBI, http://www.ncbi.nlm.nih.gov/genomes/FLU/) [30] and the EpiFlu^™^ Database from the Global Initiative on Sharing Avian Influenza Data (GISAID, http://platform.gisaid.org/) [31].

### Sequence Analysis

Protein translation and multiple sequence alignment were performed using BioEdit (Version 7.2.5) [32]. The position-specific amino acid compositions were plotted on graphs using WebLogo 3 [33]. A phylogenetic tree was inferred using the Neighbor-Joining method with 1,000 replicates as implemented in MEGA6 [34]. A mutual information (MI) analysis was performed on Mutual Information Server To Infer Coevolution (http://mistic.leloir.org.ar/) to detect coupled mutations [35].

### Homology Modeling

A homology model of the PB2 protein for A/Taiwan/4-CGMH2/2014(H7N9) was constructed using SWISS-MODEL [36], which was visualized using PyMOL [37]. Electrostatic surface potentials were predicted using Chimera [38] by applying Coulomb’s law, and the surfaces were colored from −4 kT/e (red) to 4 kT/e (blue). A/little yellow-shouldered bat/Guatemala/060/2010(H17N10) (PDB ID 4WSB) was used as the template to construct a plausible conformation for the entire PB2 protein.

### Site-Directed Mutagenesis

The RNP complex coding sequences derived from A/Anhui/1/2013(H7N9) were synthesized using DNA synthesis (IDT or GeneArt), cloned into the pcDNA3.1/myc-HisA and pFLAG-CMV5.1 vectors, and labeled H7N9-PB1, -PB2, -PA, and -NP. Site-directed mutagenesis was performed using pFLAG-CMV5.1-H7N9-PB2 to generate -K627E, -V139I&K627E, -K191E&K627E, -V511I&K627E, -M535L&K627E, -N559T&K627E, -M570I&K627E, -Q591K&K627E, -I647V&K627E, -M676V&K627E, and -D701N&K627E plasmids by mutagenic primer sets (S2 Table). Plasmid polI-CAT-RT (pPOLI-CAT-RT) was provided by Dr G. Brownlee (Sir William Dunn School of Pathology, Oxford, UK).

### Chloramphenicol acetyl transferase enzyme-linked immunosorbent assay

RNP activity was measured using a chloramphenicol acetyl transferase enzyme-linked immunosorbent assay (CAT ELISA; Roche, Indianapolis, IN, USA). First, 293T cells were cotransfected with 1 μg of the H7N9 RNP components cloned into pcDNA3.1. Concurrently, a plasmid containing a reporter gene (CAT) flanked by the viral promoters (pPOLI-CAT-RT) was transfected into the cells. The total cell lysate was extracted 48 h after transfection using the 1× lysis buffer provided in the CAT ELISA kit. After quantifying the protein content, each protein sample was diluted to 5 μg/μL and serially diluted using the lysis buffer. Subsequently, the sample was assayed according to the manufacturer’s instructions to determine the CAT levels.

## Results

### Genetic Characteristics of Taiwanese H7N9 Genomes

Table 1 lists the amino acid variations among the five H7N9 virus genomes reported in Taiwan. TW1/2013, TW2/2013, and TW2/2014 are from the first, second, and third patients, reported in April and December 2013 and April 2014, respectively. CGMH1 and CGMH2 are two genomes from the same specimen of the fourth patient and represent viruses from egg passage 1 and MDCK passage 2, respectively.

**Table 1:** Amino acid variations of the four Taiwanese H7N9 genomes since 2013.

Gene	AA	Patient ID					A/	Avian H7N9 Viruses
	Pos	1^st^	2^nd^	3^rd^	4^th^		Anhui/	
		TW1/	TW2/	TW2/	cg	cg	1/	
		2013	2013	2014	1	2	2013	
HA	186	L	L	**I**	**I**	.	L	L(400), **I(60)**
	235	**P**	L	L	L	.	L	L(396), **Q(64)**
	270	R	**K**	R	R	.	R	R(457), **K(3)**
	299	**N**	S	S	S	.	S	S(460)
	394	L	**I**	L	L	.	L	L(460)
	396	E	E	**A**	**A**	.	E	E(418), **A(22), D(20)**
	499	S	S	**R**	**R**	.	S	S(386), **N(32), D(22), T(19), A(1)**
NA	16	I	**T**	**T**	I	**T**	I	I(392), **V(34), T(20)**
	68	**N**	T	T	T	.	T	T(446)
	75	R	**K**	R	R	.	R	R(427), **K(17)**
	110	V	V	**I**	**I**	.	V	V(445)
	242	S	S	**P**	**P**	.	S	S(422), **P(24)**
	322	N	N	**S**	**S**	.	N	N(292), **T(140), S(14)**
	335	D	**V**	D	D	.	D	D(442)
PB2	191	K	**E**	**E**	**E**	.	K	**E(387)**, K(49), **T(1)**
	473	M	**V**	M	M	.	M	M(415), **V(22)**
	489	S	S	**C**	**C**	.	S	S(437)
	511	V	**I**	**I**	**I**	.	V	V(271), **I(166)**
	535	M	**L**	**L**	**L**	.	M	M(273), **L(164)**
	559	N	**T**	**T**	**T**	.	N	**T(388)**, N(48), **A(1)**
	570	M	**I**	**I**	**I**	.	M	**I(306)**, M(130)
	627	K	K	**E**	**E**	.	K	**E(434)**, K(2)
	647	I	**V**	**V**	**V**	.	I	I(269), **V(168)**
	701	D	D	**N**	**N**	.	D	D(435), **N(1)**
PB1	215	R	R	**K**	**K**	.	R	R(419), **K(15)**
	372	M	M	**I**	**I**	.	M	M(386), **L(35), I(11), V(1)**
	515	S	S	**P**	**P**	.	S	S(434)
	586	K	K	K	**R**	K	K	K(431), **R(3)**
	632	**A**	V	V	V	.	V	V(433), **A(1)**
PB1-F2	23	S	S	**N**	**N**	.	S	S(359), **N(10)**
	57	Y	**C**	Y	Y	.	Y	Y(384), **C(22), S(18), F(13)**
PA	61	T	T	**I**	**I**	.	T	T(389), **I(43), A(1)**
	268	**F**	L	L	L	.	L	L(427), **F(5), I(1)**
	308	I	I	**V**	**V**	.	I	I(429), **V(4)**
	409	N	N	**S**	**S**	.	N	N(336), **S(94)**
	716	R	R	**S**	**S**	.	R	R(342), **K(43), G(26), S(21)**
PA-X	194	L	**P**	**P**	**P**	.	L	**P(260)**, L(131), **Q(4)**
	198	K	K	**R**	**R**	.	K	K(350), **R(49)**
NP	119	I	I	**T**	**T**	.	I	I(435)
	239	M	**V**	M	M	.	M	M(428), **V(5), I(2)**
M1	82	N	**T**	N	N	.	N	N(446), **T(4)**
	150	T	T	**I**	**I**	.	T	T(447), **I(3)**
M2	10	**L**	**L**	P	P	.	P	P(278), **L(166)**
	29	A	A	**T**	**T**	.	A	A(444)
NS1	67	R	**Q**	R	R	.	R	R(455), **D(3), K(1)**
	114	S	**P**	S	S	.	S	S(450), **P(5), G(3), I(1)**
	213	P	**S**	P	P	.	P	P(458), **S(1)**

HA Q235L (H7 numbering, or Q226L in H3) is a receptor-binding site for human [42]. NA 68 (both H7 and H3 numbering) at neuraminidase stalk [58] to affect NA activity. PB2 E627K increases virus replication in mammalian cells [13, 14, 17]. PB2 D701N enhances transmission in guinea pigs [9]. PA position 61 shares the same residue as in PA-X (N-terminal 191-aa), and is not repeated in PA-X section. TW1/2013 (A/Taiwan/1/2013) was collected from the 1^st^ H7N9 patient (sputum, passage E1) in Taiwan in April 2013; TW2/2013 (A/Taiwan/3/2013) was collected from the 2^nd^ patient (specimen source and passage unknown) in December 2013; TW2/2014 (A/Taiwan/2/2014, specimen origin unknown, passage E1) was collected from the 3^rd^ patient in April 2014; cg1 (CGMH1 in text, sputum, egg passage 1) and cg2 (CGMH2 in text, sputum, MDCK passage 2) were collected from the 4^th^ and latest patient reported in this study. A dot is used for cg2 if the amino acid residue is the same as in cg1. Avian H7N9 viruses were provided for reference. A/Anhui/1/2013 is the vaccine candidate, and amino acids different from the vaccine strain were bolded.

With 10 amino acid substitutions, PB2 had the highest mutation (Table 1), followed by HA and NA, each with seven substitutions. In addition, PB1 and PA of these H7N9 genomes each had five positions exhibiting heterogeneity. PB1-F2, a 90-amino acid product, was alternatively translated from frame 2 of PB1 [39]. PA-X, a 252-amino acid product, was translated through a frame-shifting mechanism [40], in which the first 191-amino acids were the same as PA (coding sequence 1..573) and the remaining 61-amino acids were from frame 2 of PA (coding sequence 575..760). Although only two amino acid substitutions were observed in PA-X (Table 1), an additional substitution at position 61 of PA-X was omitted from the table because PA-X and PA share the first 191-amino acid segment. No variation was observed in NS2 among these Taiwanese genomes.

Among the 46 positions listed in Table 1, 24 had amino acid substitutions from the first and second genomes, and 33 from the second to the third genome, indicating that the substitutions were frequent. Thirteen of the 33 substitutions between the second and third genomes changed their respective amino acid residues to the original residues in TW1/2013. TW2/2014 and CGMH2 (MDCK passage 2) had identical genomes at the amino acid level. The two CGMH genomes, however, differed at two positions, NA 16 I vs T and PB1 586 R vs K. A number of the amino acid residues listed in Table 1 are well-known for their virulence and have been annotated [9, 13, 14, 17, 41–43]. In particular, both PB2 K627E and D701N were observed only in TW2/2014 and the two CGMH genomes, in which 627E is characteristic of the avian species.

Amino acid residues for the H7N9 vaccine candidate A/Anhui/1/2013 were included in Table 1 for reference. Also included were the amino acid compositions for avian H7N9 viruses that we downloaded from NCBI at these positions. The mutations from A/Anhui/1/2013 were bolded, which were mostly seen in the two Taiwanese isolates from the 3^rd^ and 4^th^ patients in 2014, suggesting that the H7N9 viruses have drifted away from the vaccine candidate. Many avian H7N9 viruses exhibited diverse genetic makeups at the listed amino acid positions in Table 1. In particular that PB2 191, 559, 570, 627 and PA-X 194 (underlined in Table 1) each displayed a dominant residue different from the ones in A/Anhui/1/2013. HA Q226L (Q235L in H7 numbering) was seen in the 2^nd^ through the 4^th^ Taiwanese patients. Even in avian H7N9 viruses, this mutation affecting receptor-binding was already seen in 396 of 460 HA sequences we examined. It was mentioned that two PB2 mutations Q591K and D701N could enhance polymerase activity of avian viruses in human 293T cells [27]. PB2 591 remained Q for all Taiwanese isolates, A/Anhui/1/2013, and all avian H7N9 viruses that we analyzed (data not shown). While PB2 701D was seen in A/Anhui/1/2013, a mutation to N was observed in two out of four Taiwanese isolates. On the contrary, there was only one displaying D701N among the avian H7N9 viruses.

### Human H7N9 Residues on Human–Avian Signature Locations

Over 140 H7N9 genomes recorded between late March 2013 and late April 2015 were retrieved from the NCBI and GISAID databases to comprehensively explore the H7N9 amino acid transitions. In a previous study, we reported 47 species-associated signatures that potentially marked a genetic boundary at which an avian influenza A virus can efficiently transmit to or replicate in humans [29]. Table 2 lists these residues and the associated amino acid compositions for the human H7N9 viruses. The residues for CGMH2 and A/Shanghai/2/2013 were included for comparison. HA and NA positions are missing in Table 2 because they were excluded in our previous studies [28, 29]. Avian-like residues were identified in most of these signature positions for the H7N9 virus, indicating that it is avian in origin. A few exceptions included PB2 627 and PA 100, 356, and 409 because they exhibited human-like residues K, A, R, and N, respectively. Conversely, CGMH2 included only two human-like residues PA 100A and 356R. In addition, human H7N9 exhibited a truncated NS1 that was a 217-amino acid segment, thus missing the signature at position 227.

**Table 2:** Amino acid compositions at 47 species-associated signature positions for H7N9 viruses.

Gene	Pos	Avian	Human	H7N9	CGMH2	Shanghai/2/2013
PB2	44	A	S	A(147)	A	A
	199	A	S	A(146), S(1)	A	A
	271	T	A	T(147)	T	T
	475	L	M	L(147)	L	L
	567	D	N	D(147)	D	D
	588	A	I	A(139), V(8)	A	A
	613	V	T	V(149)	V	V
	**627**	E	**K**	**K**(100), E(45)	E	**K**
	702	K	R	K(147)	K	K
PB1	327	R	K	R(137)	R	R
	336	V	I	V(137)	V	V
PA	28	P	L	P(139)	P	P
	55	D	N	D(139)	D	D
	57	R	Q	R(136), Q(3)	R	R
	**100**	V	**A**	**A**(99), V(37), I(3)	**A**	**A**
	225	S	C	S(138), C(1)	S	S
	268	L	I	L(135), F(4)	L	L
	**356**	K	**R**	**R**(126), K(13)	**R**	**R**
	404	A	S	A(139)	A	A
	**409**	S	**N**	**N**(125), S(14)	S	**N**
	552	T	S	T(139)	T	T
NP	16	G	D	G(140)	G	G
	33	V	I	V(140)	V	V
	61	I	L	I(137), M(3)	I	I
	100	R	V	R(139), K(1)	R	R
	109	I	V	I(140)	I	I
	214	R	K	R(140)	R	R
	283	L	P	L(140)	L	L
	293	R	K	R(140)	R	R
	305	R	K	R(140)	R	R
	313	F	Y	F(139), L(1)	F	F
	357	Q	K	Q(139), R(1)	Q	Q
	372	E	D	E(140)	E	E
	422	R	K	R(140)	R	R
	442	T	A	T(140)	T	T
	455	D	E	D(140)	D	D
M1	115	V	I	V(143)	V	V
	121	T	A	T(143)	T	T
	137	T	A	T(143)	T	T
M2	11	T	I	T(142)	T	T
	20	S	N	S(141), N(1)	S	S
	57	Y	H	Y(142)	Y	Y
	86	V	A	V(142)	V	V
	93	N	S	N(142)	N	N
NS1	81	I	M	I(141)	I	I
	227	E	R	Delete	Delete	Delete
NS2	107	L	F	L(142)	L	L

Positions shown in bold represent H7N9 viruses carrying human-characteristic amino acids.

### Position-specific Amino Acid Variations for H7N9

**Fig 1:**
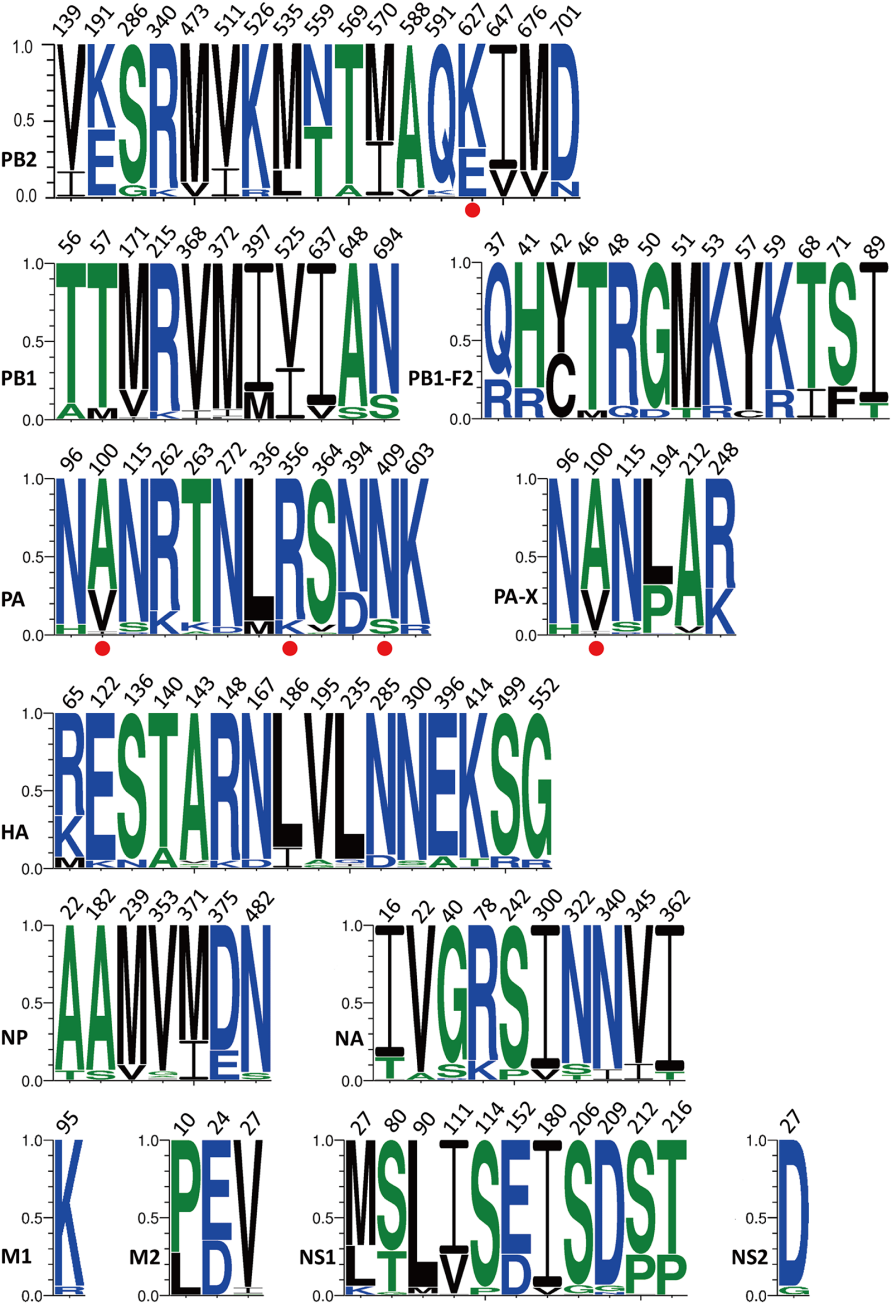
Web logos for H7N9 genome displaying at least 7 substitutions. Positions accompanied by red dots are among the 47 species-associated signature positions listed in Table 2. The leading three logos for PA-X and PA are identical, as the two proteins share the 191 amino acids at the N terminal. Sequence counts used in each of these logos are included in the parentheses as PB2 (147), PB1 (137), PB1-F2 (134), PA (139), PA-X (132), HA (142), NP (140), NA (141), M1 (143), M2 (142), NS1 (141), NS2 (142). The Y-axis shows the residue frequency for each residue, which adds up to 1.0.

Only four human-avian signature positions developed human-like residues that dominated the H7N9 population (Table 2). No human-like residues were observed in other signature positions, suggesting that some nonsignature positions contribute to human infections. We scanned the entire set of 12 protein alignments and summarized the compositions for the positions exhibiting genetic diversity. Fig 1 illustrates the web logos for 104 positions of seven or more substitutions that represent the most visible amino acid variations. PA 96, 100, and 115, and PA-X 96, 100, and 115 exhibited identical logos because both had the same open reading frame for their initial 190 amino acids.

Only four (PB2 627, PA 100, 356 and 409, red dots in Fig 1) of the 36 RNP-related (PB2, PB1, PA, and NP) signature positions exhibited visible variations (Fig 1 and Table 2). In other words, more amino acid positions were free to evolve as nonsignature positions compared with the signature positions associated with the human–avian boundary. PB2 exhibited the most abundant 17 variations, followed by HA (16), PB1-F2 (13), PB1 (11), PA (11), NS1 (11), and NA (10). Of these 104 logos, 62 belonged to the RNP genes.

### Amino Acid Cosubstitution in H7N9 PB2 Proteins

We further explored the interlacing of the 17 PB2 substitutions (Fig 1). Table 3 summarizes the multiple sequence alignment for these substitutions. PB2 sequence of A/Shanghai/2/2013 was used as the baseline for displaying residue changes. Sequences with no or only one substitution at these 17 locations were excluded for brevity. The remaining 78 sequences were divided into three temporal groups based on influenza seasonality: season I from March to September 30, 2013 (20 viruses), season II from October 1, 2013 to September 30, 2014 (40 viruses), and season III after October 1, 2014 (18 viruses). Within each season, the order of appearance was arbitrarily selected to enhance the illustration of the substitution patterns. In particular, amino acid substitutions cooccurring with K627E were bolded, and their counts were summarized (Table 3).

**Table 3:** Selected PB2 amino acid positions showing cosubstitution with K627E.

Strain Name \ PB2 Position	Date	139*	191*	286	340	473	511*	526	535*	559*	569	570*	588	591*	627	647*	676*	701*
A/Shanghai/2/2013	Mar 5	V	K	S	R	M	V	K	M	N	T	M	A	Q	K	I	M	D
			Strains isolated between March 28 and September 30, 2013 –season I															
A/Nanjing/1/2013	Mar 28													**K**	E			
A/Jiangsu/1/2013	Apr 20													**K**	E			
A/Jiangsu/2/2013	Apr 20													**K**	E			
A/Zhejiang/DTID-ZJU08/2013	Apr?													**K**	E			
A/Zhejiang/2/2013	Apr 3														E			**N**
A/Zhejiang/DTID-ZJU01/2013	Apr 3														E			**N**
A/Jiangsu/05/2013	Apr 8														E			**N**
A/Jiangsu/08/2013	Apr 12														E			**N**
A/Jiangsu/03/2013	Apr 6							**R**							E			**N**
A/Zhenjiang/1/2013	Apr 7							**R**							E			**N**
A/Zhejiang/DTID-ZJU02N/2013	Apr?							**R**							E			**N**
A/Zhejiang/DTID-ZJU02R/2013	Apr?							**R**							E			
A/Beijing/3/2013	Apr 19								T						E			
A/Changsha/2/2013	Apr 29						**I**								E			
A/Beijing/1/2013	Apr 16		**E**												E			
A/Beijing/01-A/2013	Apr 16		**E**							**T**		L			E			
A/Shanghai/JS01/2013	Apr 3		**E**				**I**		**L**	**T**		**I**			E	**V**		
A/shanghai/05/2013	Apr 2		**E**				**I**		**L**	**T**		**I**			E	**V**		
A/Nanjing/3/2013	Apr 19		E				I		L	T		I				V		
A/Shanghai/13/2013	Apr 10		E				I		L	T		I				V		
A/Huizhou/01/2013	Aug 8	I	E							T		I					V	
A/Guangdong/1/2013	Aug 10	I	E							T		I					V	
Co-substitution / substitution frequency (season 1 subtotal)		0 /2	4 /8	0 /0	0 /0	0 /0	3 /5	4 /4	2 /5	3 /7	0 /0	2 /7	0 /0	4 /4	n/a	2 /4	0 /2	7 /7
			Strains isolated between October 1, 2013 and September 30, 2014 –season II															
A/Zhejiang/22/2013	Oct 14									**T**					E			
A/Guangdong/02/2013	Nov 4	**I**	**E**							**T**		**I**		**L**	E		**V**	
A/Shenzhen/SP116/2014	Mar 15	**I**	**E**							**T**		**I**			E		**V**	
A/Hong Kong/8113530/2014	Mar 17	**I**	**E**							**T**		**I**			E		**V**	
A/Hong Kong/2212982/2014	Jan 28	**I**	**E**							**T**		**I**			E		**V**	**N**
A/Shenzhen/SP113/2014	Mar 12	**I**	**E**							**T**		**I**			E		**V**	**N**
A/Jilin/10117/2014	Feb 19		**E**				**I**		**L**	**T**		**I**			E	**V**		**N**
A/Taiwan/2/2014	Apr 24		**E**				**I**		**L**	**T**		**I**			E	**V**		**N**
A/Taiwan/4-CGMH2/2014	Apr 25		**E**				**I**		**L**	**T**		**I**			E	**V**		**N**
A/Shenzhen/SP16/2014	Jan 18	**I**	**E**	**G**						**T**	**A**	**I**			E		**V**	**N**
A/Shenzhen/SP60/2014	Jan 30	**I**	**E**	**G**						**T**	**A**	**I**			E		**V**	**N**
A/Shenzhen/SP44/2014	Jan 23	**I**	**E**	**G**						**T**	**A**	**I**			E		**V**	
A/Hong Kong/470129/2013	Nov 30	I	E	G						T	A	I					V	
A/Hong Kong/5942/2013	Nov 30	I	E	G						T	A	I					V	
A/Hong Kong/734/2014	Jan 7	I	E	G						T	A	I					V	
A/Shenzhen/SP-Z93/2014	Jan 11	I	E	G						T	A	I					V	
A/Shenzhen/SP17/2014	Jan 20	I	E	G						T	A	I					V	
A/Shenzhen/SP26/2014	Jan 20	I	E	G						T	A	I					V	
A/Shenzhen/SP38/2014	Jan 22	I	E	G						T	A	I					V	
A/Shenzhen/SP49/2014	Jan 25	I	E	G						T	A	I					V	
A/Shenzhen/SP58/2014	Jan 25	I	E	G						T	A	I					V	
A/Guangdong/05/2013	Dec 17	I	E							T		I		L			V	
A/Taiwan/3/2013	Dec 27		E			V	I		L	T		I				V		
A/Shanghai/01/2014	Jan 3		E			V	I		L	T		I				V		
A/Shanghai/PD-02/2014	Jan 17		E			V	I		L	T		I				V		N
A/Shenzhen/SP118/2014	Mar 18		E			V	I		L	T		I				V		
A/Taiwan/1/2014	Apr 21		E			V	I		L	T		I				V		
A/Shanghai/Mix1/2014	Jan 3		E				I		L	T		I				V		
A/Shanghai/PD-01/2014	Jan 17		E				I		L	T		I				V		
A/Zhejiang/LS01/2014	Feb 8		E				I		L	T		I				V		
A/Guizhou/01502/2014	Jan 8		E				I			T		I				V		
A/Hong Kong/3263/2014	Feb 12	I	E							T		I					V	
A/Shenzhen/SP75/2014	Feb 15	I	E							T		I					V	
A/Shenzhen/SP48/2014	Jan 23	I	E							T		I					V	
A/Shenzhen/SP62/2014	Feb 5	I	E							T		I					V	
A/Shenzhen/SP-W1/2014	Jan 14	I	E							T		I					V	
A/Shenzhen/SP126/2014	Mar 23		E							T								
A/Shenzhen/SP139/2014	Apr 2		E							T								
Co-substitution / substitutionfrequency (season 2 subtotal)		8 /23	11 /37	3 /12	0 /0	0 /5	3 /12	0 /0	3 /11	12 /38	3 /12	11 /35	0 /0	1 /2	n/a	3 /12	8 /23	7 /8
			Strains isolated between October 1, 2014 and September 30, 2015 –season III															
A/Jiangsu/98342/2014	Nov 17		**E**				**I**		**L**	**T**		**I**			E	**V**		
A/Env/Xinjiang/98679/2014	Nov 28		**E**			**V**	**I**		**L**	**T**		**I**			E	**V**		
A/Xinjiang/05845/2015	Jan 8		**E**			**V**	**I**		**L**	**T**		**I**		**K**	E	**V**		
A/Zhejiang/17/2014	Nov 25		**E**			**V**	**I**		**L**	**T**		**I**			E	**V**		
A/Hong Kong/8130773/2015	Feb 22		**E**		**K**					**T**			**V**		E			
A/Guangdong/15SF010/2015	Jan 6		**E**		**K**					**T**			**V**		E			
A/Xinjiang/05916/2014	Dec 25		E			V	I		L	T		I				V		
A/Jiangsu/06306/2014	Dec 27		E			V	I		L	T		I				V		
A/Quzhou/2/2015	Apr 23		E			V	I		L	T		I				V		
A/Quzhou/1/2015	Apr 25		E			V	I		L	T		I				V		
A/Guangdong/02496/2014	Nov 27		E		K					T			V					
A/Hong Kong/8194273/2014	Dec 25		E							T								
A/XinjiangBintuan/99117/2014	Oct 25		E		K					T			V					
A/Guizhou/03240/2015	Jan 9		E		K					T			V					
A/Guangdong/15SF018/2015	Jan 12		E		K					T			V					
A/Fujian/09273/2015	Jan 12		D		K					T			V					
A/British Columbia/1/2015	Jan 16		E							T								
A/Hong Kong/2550/2015	Jan 23		E		K					T			V					
Co-substitution / substitutionfrequency (season 3 subtotal)		0 /0	6 /18	0 /0	2 /8	3 /7	4 /8	0 /0	4 /8	6 /18	0 /0	4 /8	2 /8	1 /1	n/a	4 /8	0 /0	0 /0
Co-substitution / substitution frequency (all-time)		8* /25	21*/63	3 /12	2 /8	3 /12	10*/25	4 /4	9* /24	21*/63	3 /12	17*/50	2 /8	6* /7	n/a	9* /24	8* /25	14*/15

A/Taiwan/4-CGMH2/2014 is the 4^th^ Taiwanese H7N9 human infection reported and sequenced in this study. Substitutions co-emerged with 627E are bolded. Amino acid positions with asterisk (*) displayed 6 or more co-substitutions with 45 strains having 627E. These are the same 10 mutations used in RNP activity assay for assessing their compensatory effect with K627E in Fig 3.

Along with PB2 K627E, four strains isolated early in season I exhibited Q591K. Such a cosubstitution reemerged only in a recent Xinjiang strain on January 8, 2015. Moreover, 14 viruses exhibited another covarying position D701N with K627E observed in seasons I and II but missing in season III. Although no cooccurrence of Q591K, K627E, and D701N was observed, other amino acid changes coemerged with K627E at various degrees. For example, V139I, K191E, V511I, M535L, N559T, M570I, I647V, and M676V each had cosubstitutions ranging from 8 to 21 instances with respect to K627E. Although S286G, R340K, M473V, K526R, T569A, and A588V were observed (Table 3), their association with K627E was seen in four or fewer instances.

Certain PB2 sites (Table 3) exhibited cosubstitutions regardless of the PB2 627 residue they carried. One example is the combination {V139I, S286G, T569A, M676V}, observed only in season II (November 2013–January 2014) in 12 strains from Shenzhen and Hong Kong; among them, three carried 627E and nine carried 627K. Another example is the combination {M473V, V511I, M535L, I647V} appearing in 12 different strains—five in season II including A/Taiwan/3/2013 and A/Taiwan/1/2014, and seven in season III. Only three of these 12 strains were associated with 627E. {K191E, N559T, M570I} is a most appearing combination in 50 or more sequences in Table 3. The trio emerged in April 2013 in early stage of the outbreak and stayed in the H7N9 population in almost the entire seasons II and III, except that M570I seemed to fade out in half of the strains collected in season III.

The alignment containing 79 PB2 sequences and 17 amino acid positions from Table 3 was used to infer their coupled mutations using MI. S1 Fig shows the covariation network for these 17 PB2 positions, including the predicted MI scores depicting the degree of covariation between any of the paired mutations. The same alignment, as well as the 79 full-length PB2 sequences of 759-aa long were used to infer two phylogenetic trees shown in S2 Fig, on which the 17 amino acid mutations outlined in Table 3 were labeled on tree branches for tracing their evolutionary pathway.

### CGMH2 PB2 Protein Stereography

**Fig 2:**
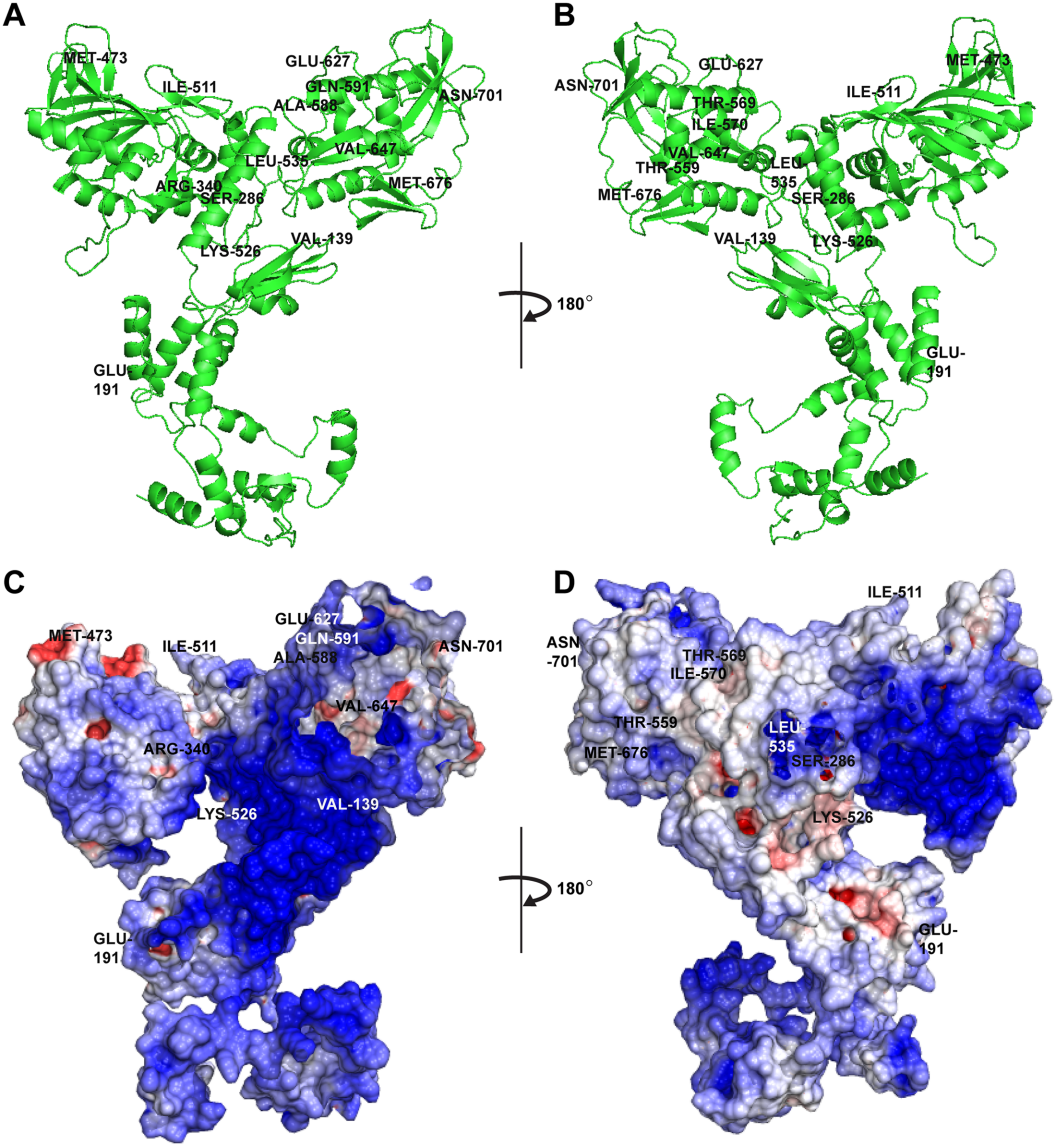
Stereography of influenza A H7N9 PB2 residue-varying positions listed in Table 3. (A and B) Simulated H7N9 PB2 stereography for A/Taiwan/4-CGMH2/2014(H7N9) viral PB2 is shown in the ribbon mode. (C and D) The distribution of electrostatic potential on the protein surface (blue = relative positive charge; red = relative negative charge). Protein modeling was performed using SWISS-MODEL by applying Coulomb’s law in Chimera according to the full-length PB2 of A/little yellow-shouldered bat/Guatemala/060/2010(H17N10) (PDB ID 4WSB).

As listed in Table 3, 42 of the listed H7N9 viruses exhibited a human-like signature K at position 627, and 36 retained an avian-like E signature. This suggests the existence of other mutations, which would possibly compensate for the K627E change, enabling this avian virus to infect humans. To resolve the spatial correlation among the amino acid positions that exhibited covariations with PB2 627, a simulated CGMH2 PB2 structure was modeled based on the full-length PB2 of A/little yellow-shouldered bat/Guatemala/060/2010(H17N10) (PDB ID 4WSB). This bat influenza H17N10 virus was chosen because it is the latest and the only full-length PB2 being resolved thus far [44], comparing with the other commonly used avian influenza H5N1 PB2 C-terminal domain (CTD) structure (PDB ID 3KC6) of 204-aa long covering only positions 538 to 741 of a full-length PB2 protein. Although this bat virus shares only 67.6% identity with the full-length PB2 of CGMH2, the simulated structure within CTD by using 4WSB was found qualitatively comparable with the one simulated by 3KC6 (data not shown). Similar to the findings of a previous report [9], the residues 591Q and 627E shared a surface (Fig 2). Spatially dispersed 139V, 191E, 473M, 511I, 526K, 647V, and 701N, which were distant from 627E, are illustrated in Fig 2C. Fig 2D illustrates the other side of the simulated protein structure, on which residues 569T and 570I were right next to each other and within the 627 domain.

### RNP Activity for Covarying PB2 Amino Acids in Human H7N9 Virus

**Fig 3:**
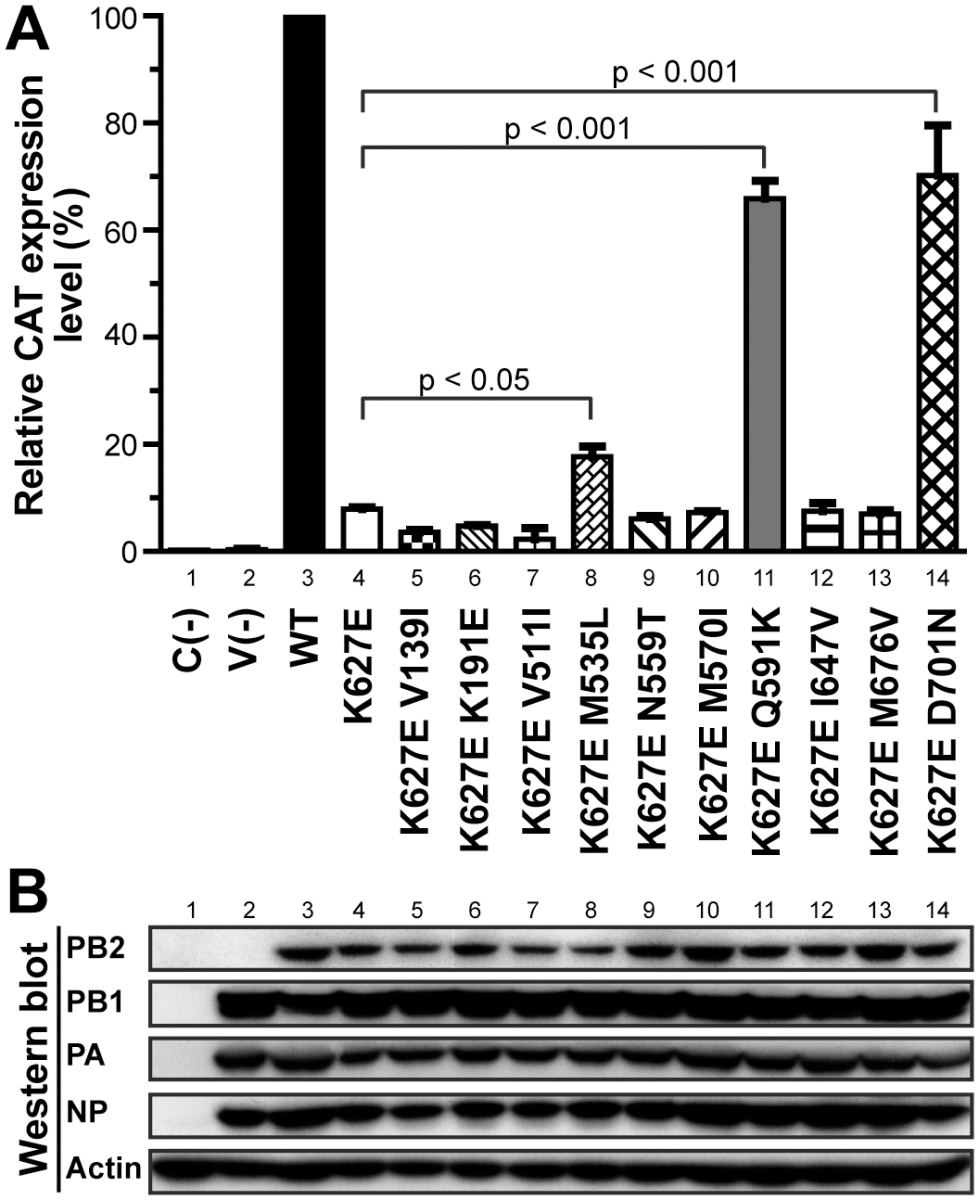
Statistics for the RNP activity assay of the influenza A H7N9 PB2. (A) RNP activity for the H7N9 virus in 293T cells. The RNP complexes exhibiting combinations of the avian signature PB2 627E and its covarying residues (positions labeled with asterisks in Table 3) from the A/Anhui/1/2013(H7N9) virus were cotransfected into the 293T cells. CAT ELISA was performed 48 h after transfection to detect protein expression levels of the virus-like CAT reporter, thereby signaling the viral RNP activity. The error bars show the SEM from 3 independent experiments. Two-way ANOVA and the GraphPad Prism Software were used to statistically support the observed disparity. (B) Each component of the viral RNP complex and NP was detected using Western blotting.

A CAT reporter RNP activity assay was performed to evaluate the effects of the PB2 K627E covariations. Only 10 mutations (amino acid positions with asterisks in Table 3) displaying at least six cosubstitutions with the 36 strains having K627E were tested. As shown in Fig 3, the RNP activity of PB2 627E (a signature characteristic to avian species) markedly reduced to 7.9% in the human cells. The Q591K and D701N residues accompanying K627E considerably restored the RNP activity back to 65.9% and 70.2%, respectively (both with *P* < 0.001). M535L is another mutation exhibiting the compensatory effect with 627E (17.7%, *P* < 0.05). However, the other seven amino acid substitutions that covaried with K627E exhibited no such effect.

## Discussion

The mutation Q226L of influenza HA protein was reported to increase binding to receptors in human upper airway [24]. In 142 H7N9 HA sequences we examined in this study, 133 already showed L at this position. Only 4 strains displayed Q, including A/Shanghai/1/2013, A/Shanghai/05/2013, A/Shanghai/JS01/2013 and A/Nanjing/5/2013, all were isolates from April 2013 or earlier. These demonstrated these human-isolated H7N9 viruses have already evolved to fit human receptors in these three seasons. Interestingly, we found 396 out of 460 avian H7N9 viruses already carried this mutation (Table 1). This raises the concern for avian H7N9 viruses to continue infecting human.

As presented in Table 1, the two CGMH viruses had identical genome sequences except at NA 16 and PB1 586. The two genomes were sequenced from the same specimen cultured using different culture systems. This result is consistent with those of other studies, in which a sequencing discrepancy was observed among different viruses isolated from a single patient. Lin et al. [45] determined the genome sequences from three samples of the first Taiwanese patient with an H7N9 infection: two samples each from a sputum and throat swab on one given day, and a third from a throat swab on another day. At PB2 627, a virulence factor for efficient viral replication, the sputum specimen produced a residue K for promoting replication in mammalian cells, whereas the other two remained E, as observed in most avian species. Through a pyrosequencing assay, Mok et al. [46] revealed that both residues R and K developed at an oseltamivir-resistant marker NA 292 (equivalent to position 289 in H7N9 numbering) as quasispecies; this was also observed in the sputum specimen of the first Taiwanese H7N9 patient. A change from R to K renders the virus resistant to oseltamivir. Examples such as these demonstrate how this quasispecies exhibited intrahost genome variability among samples collected from different tissues, on different days of disease progression, or when using different laboratory host systems. RNA viruses, in particular the influenza A viruses, are known to mutate frequently. The missing fidelity during genome replication could explain the sequencing discrepancies observed in these works. Whether such mutations were simply spontaneously and randomly produced or could be the result of different selection pressures such as tissues, culture systems, or host adaptation during disease progress, would require further investigation.

Although the two influenza virus surface genes HA and NA were subjected to host selection and were therefore generally more variable than the internal genes, the H7N9 RNP genes seemed to exhibit higher genetic diversity than did HA and NA; this can be seen in Fig 1, where numerous RNP residues exhibited more divided logos, suggesting the importance of polymerase genes in the evolution of the H7N9 virus for host adaptation. Another possible source for the observed genetic heterogeneity is the diverse avian genomes inherited by the H7N9 viruses. The novel H7N9 virus inherited six of its internal genes from the avian H9N2 viruses. Chen et al. [18] compared the H7N9 PB2 sequences with 287 avian H9N2 PB2 sequences and reported that their percent identities were markedly variable from as low as 81.4% to as high as 99.2%, indicating the intrinsic genetic diversities in H7N9 PB2 from various bird populations. Additional evidence for PB2 diversity was obtained in four of the earliest H7N9 human isolates reported in two weeks in Shanghai (A/Shanghai/1/2013–A/Shanghai/4/2013); in these isolates, 6 of the 759 amino acid positions had already exhibited substitutions (data not shown). These observations demonstrate that the novel H7N9 viruses inherited PB2 from multiple avian origins.

In addition to mutations at PB2 627, mutations at PB2 591 and 701 were strongly involved in mammalian host restriction [47]. In this study, we demonstrated that Q591K and D701N, each accompanied by K627E, restored the RNP activity (*P* < 0.001). These affirmed the finding by Mok et al. [27] that Q591K and D701N are crucial in compensating for the absence of 627K. In addition, M535L+K627E restored the RNP activity (*P* < 0.05). Although the two positions 591 and 627 were structurally close with apparent involvement in the same host factors, the residues 535 and 701 were spatially distant from 627, suggesting the involvement of a different mechanism. Of the 147 PB2 sequences we analyzed, only five had Q591K, and four of them occurred at an extremely early stage of H7N9 endemic in March and April 2013. In all, 15 D701N substitutions were observed, and none occurred after January 2014. Conversely, 23 M535L substitutions were observed in all three seasons, signifying its regular presence and potential role in the increasing H7N9 adaptation to humans.

Several other amino acids exhibited covarying patterns with K627E, which were consistent with those exhibited by Q591K, M535L, and D701N. However, the RNP activity assays suggested no correlation between the amino acid changes at these positions with PB2 K627E. Some of these substitutions, however, formed their own coevolving patterns. For example, we found {V139I, S286G, T569A, M676V}, occurring only in season II (November 2013–January 2014), in 12 strains from Shenzhen and Hong Kong, regardless of whether they were accompanied by 627E or K. Such amino acid coevolutions suggest their possible involvement in polymerase activities. We did not rule out any other coevolving residues that emerged in other H7N9 genes or across different genes. Further investigation is warranted to determine whether these changes occurred spontaneously or were correlated with host adaptability or other functions.

RNP activity is measured by a constitutional assay. Influenza viral genes PA, PB1, PB2 and NP were cotransfected into cells to express RNP complex. Meanwhile, a reporter gene, such as luciferase, CAT, or GFP flanked by viral promoters was also transfected into cells to be driven by RNP complex [48]. RNP activity assay has not only been applied in H7N9 [26, 27, 49] but also in H5N1 avian influenza viruses [6, 50, 51], 2009 pandemic H1N1 influenza virus [52–55], and seasonal H3N2 influenza virus [51, 54]. The RNP activity does not entirely correlate to the replication rate [56]. As a result, using this assay alone to fully assess the viral replication efficiency during crossing species should be considered as tentative. Nevertheless, it is a surrogate and reliable assay to examine the influence of any mutation of RNP genes on RNP activity. Moreover, PB2 is known to enter mitochondrion [57]. Other than RNP activity, the mutation K627E and other co-substitutions may affect the mitochondrion localization of PB2 protein. Such mechanism may also associate with human adaption for this virus.

Chen et al. proposed species-specific signatures that have been widely used as markers to assess the possibility of human infection from avian influenza viruses. These signatures were determined according to an analysis of 306 human and 95 avian virus genomes in 2006 [28]; these signatures were revalidated in 2009 on the basis of an analysis of over 3,000 genomes of each of the two viruses [29]. Although an avian influenza A virus can be reasonably assumed to have acquired more human signatures thereby increasing its potential to infect humans, validation of such an assumption using only sequence analysis is challenging. Although biological experiments may be useful in proving such an assumption, these experiments may confer the risk of generating a potential pandemic strain. Currently, evidence supporting a correlation between the number of species-specific signatures and the possibility of human infection is lacking. Nevertheless, analyzing human-isolated avian influenza virus genomes is crucial because they may reveal alternative positions that compensate for mutations at certain signature or nonsignature positions.

## Conclusion

Numerous human-isolated avian influenza A viruses exhibit PB2 627K, which is a human-specific signature, and strong biological evidence indicates that an E627K mutation promotes avian viral replication in mammals. However, 45 of the 147 human H7N9 isolates investigated in this study exhibited an avian signature E at this position. Additional sequence analysis suggested that 10 PB2 substitutions could potentially increase the efficiency of an avian virus in infecting humans in the absence of PB2 627K. We used a reporter assay to test all of these substitutions and showed that either Q591K, M535L, or D701N mutation increases the viral RNP activity in human cells with PB2 627E, suggesting that E627K, Q591K, M535L, and D701N are crucial markers for assessing the potential of an avian virus to infect humans, as well as for potentially increasing adaptation for the virus to gain human-to-human transmission capability in leading to a pandemic in the future.

## Supporting Information

S1 Fig PB2 amino acid sites exhibiting coupled mutations using mutual information (MI). Based on the alignment in Table 3 (79 PB2 sequences for 17 amino acid sites) using Mutual Information Server To Infer Coevolution (http://mistic.leloir.org.ar/). MI scores are labeled on the arcs connecting these amino acids. Arcs are of variable thickness to approximate the MI scores. An MI threshold of 6.5 was used according to the server default setting. K627E was found coupled with D701N, N559T, K191E, K526R and Q591K. Two nearly remote clusters were also identified, including (M473V, V511I, M535L, I647V) and (V139I, S286G, T569A, M676V).
(TIF)

S2 Fig PB2 phylogenetic trees for H7N9 viruses. PB2 sequences of H7N9 viruses listed in Table 3 were used by MEGA 6.0 to produce the Neighbor-Joining tree with 1,000 pseudo replicates. Amino acid substitutions were labeled at the tree branches to follow the trend of these 17 mutations. (A&B) The alignment contains only 17 amino acid positions that we intend to follow. (C&D) The alignment contains the entire 759-aa PB2 sequence.
(TIF)

S1 Table Primers used for H7N9 genome sequencing. (DOC)

S2 Table Primers used for site-directed mutagenesis. (DOC)
