# Supplementary figures and images for: Genomic Signatures for Avian H7N9 Viruses Adapting to Humans
Source: PLoS One. 2016 Feb 4;11(2):e0148432. doi: 10.1371/journal.pone.0148432 (PMC4742285; doi:10.1371/journal.pone.0148432)

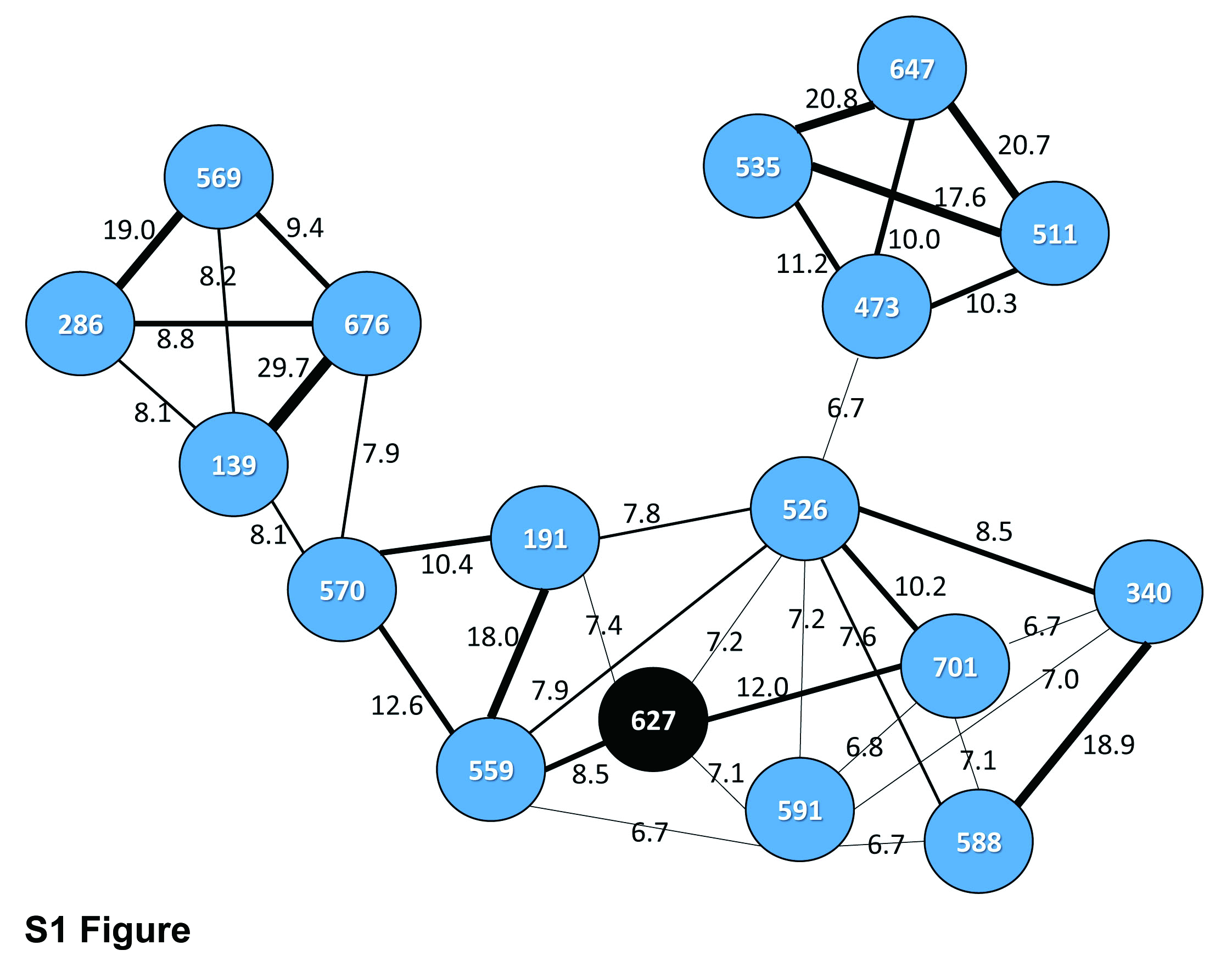

Supplement: S1 Fig — Based on the alignment in Table 3 (79 PB2 sequences for 17 amino acid sites) using Mutual Information Server To Infer Coevolution (http://mistic.leloir.org.ar/). MI scores are labeled on the arcs connecting these amino acids. Arcs are of variable thickness to approximate the MI scores. An MI threshold of 6.5 was used according to the server default setting. K627E was found coupled with D701N, N559T, K191E, K526R and Q591K. Two nearly remote clusters were also identified, including (M473V, V511I, M535L, I647V) and (V139I, S286G, T569A, M676V). (TIF) [file pone.0148432.s001.tif]

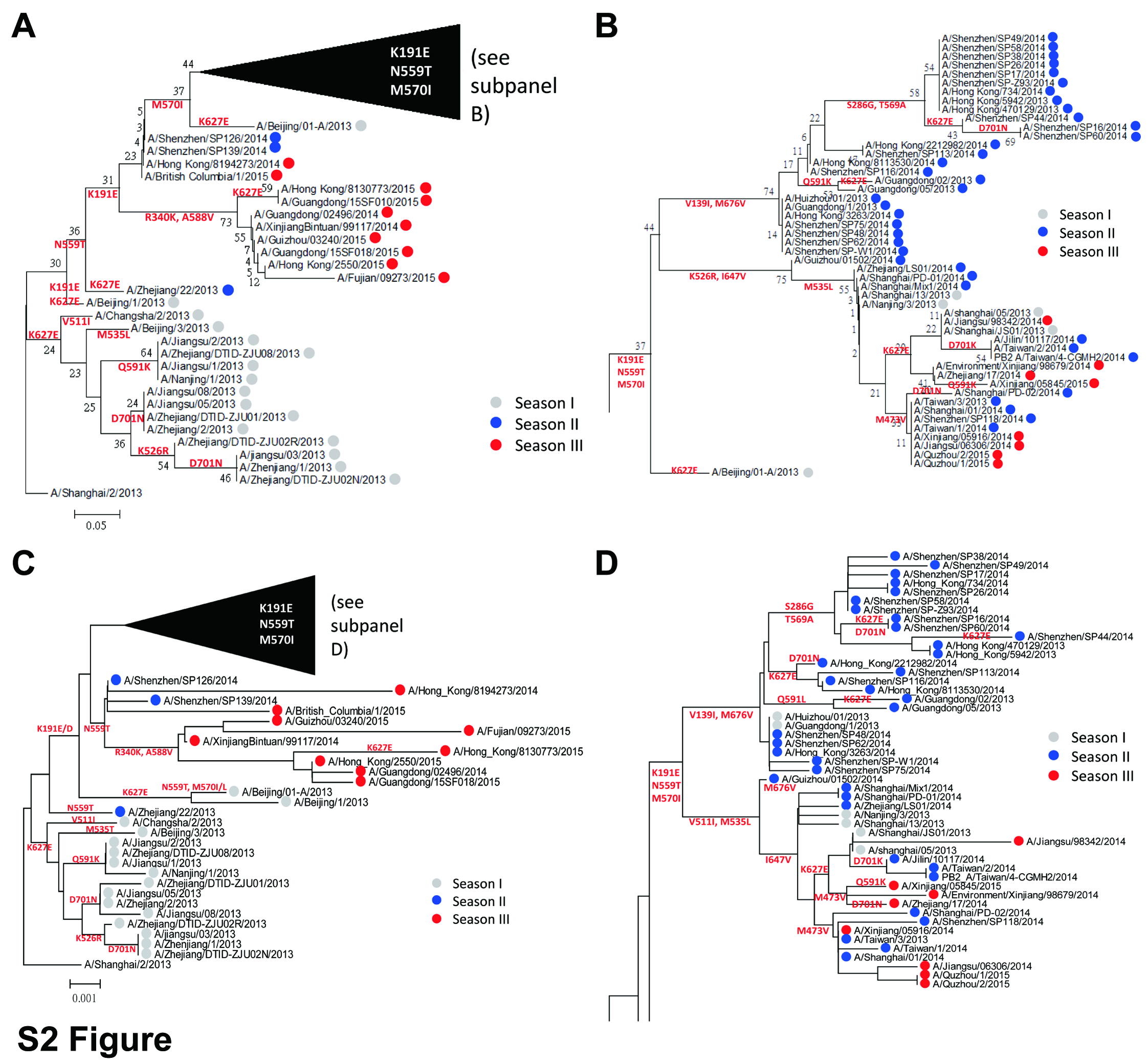

Supplement: S2 Fig — PB2 sequences of H7N9 viruses listed in Table 3 were used by MEGA 6.0 to produce the Neighbor-Joining tree with 1,000 pseudo replicates. Amino acid substitutions were labeled at the tree branches to follow the trend of these 17 mutations. (A&B) The alignment contains only 17 amino acid positions that we intend to follow. (C&D) The alignment contains the entire 759-aa PB2 sequence. (TIF) [file pone.0148432.s002.tif]
