# Supplementary material for: Genomic Signatures for Avian H7N9 Viruses Adapting to Humans
Source: PLoS One. 2016 Feb 4;11(2):e0148432. doi: 10.1371/journal.pone.0148432 (PMC4742285; doi:10.1371/journal.pone.0148432)
Supplement: S1 Table — (DOC) [file pone.0148432.s003.doc]

**S1 Table.** Primers used for H7N9 genome sequencing.

| Gene | Primer* | Sequence (5’→3’) | Annealing (°C) | Size (bp) |
| --- | --- | --- | --- | --- |
| PB2 | PB2-F | 5’-agcgaaagcaggtcaaatatattca-3’ | 50 | 2341 |
|  | PB2-2341R | 5’-agtagaaacaaggtcgtttttaaac-3’ |  |  |
| PB1 | PB1-F | 5’-atggatgtcaatccgacttt-3’ | 50 | 2274 |
|  | PB1-2274R | 5’-ctatttttgccgtctgagct-3’ |  |  |
| PA | PA-F | 5’-agcgaaagcaggtactgatcc-3’ | 50 | 2233 |
|  | PA-2233R | 5’-agtagaaacaaggtacttttttgga-3’ |  |  |
| HA | HA-F | 5’-atgaacactcaaatcctggtattc-3’ | 55 | 1683 |
|  | HA-1683R | 5’-ttatatacaaatagtgcaccgca-3’ |  |  |
| NP | NP-F | 5’-atggcgtctcaaggcaccaaacgatc-3’ | 55 | 1497 |
|  | NP-1497R | 5’-tcaattgtcatactcctctgcattg-3’ |  |  |
| NA | NA-F | 5’-atgaatccaaatcagaagattctat-3’ | 55 | 1398 |
|  | NA-1398R | 5’-ttagaggaagtactctattttagccc-3’ |  |  |
| M | M-F | 5’-atgagtcttctaaccgaggtcgaa-3’ | 55 | 982 |
|  | M-982R | 5’-ttacttcagctctatgttgaca-3’ |  |  |
| NS | NS-F | 5’-cggaattcaatggattccaatactgtgtc-3’ | 55 | 838 |
|  | NS-838R | 5’-cgggatccttaaataagctgaaacgaga-3’ |  |  |

*Primer sequences of PB1, NP, and M were referenced in the following article, whereas the primers for the other genes were kindly provided by Drs Shin-Ru Shih (PB2, PA, HA, NA ) and Rei-Lin Kuo (NS) of the Research Center for Emerging Viral Infections, Chang Gung University, Taoyuan, Taiwan.

Zhao B, Zhang X, Zhu W, Teng Z, Yu X, Gao Y, et al., Novel Avian Influenza A(H7N9) Virus in Tree Sparrow, Shanghai, China, 2013. Emerging Infectious Diseases, 2014; 20(5):850-3.
