# Supplementary material for: Genomic Signatures for Avian H7N9 Viruses Adapting to Humans
Source: PLoS One. 2016 Feb 4;11(2):e0148432. doi: 10.1371/journal.pone.0148432 (PMC4742285; doi:10.1371/journal.pone.0148432)
Supplement: S2 Table — (DOC) [file pone.0148432.s004.doc]

**S2 Table.** Primers used for site-directed mutagenesis.

| Primer Name | Sequence of mutagenic oligonucleotides (5’-3’)* | Amino acid substitution |
| --- | --- | --- |
| **K627E**mut-1F | GCAGCAGCCCCGCCGGAGCAGAGTAGGATGC | Lys-627 → Glu |
| **K627E**mut-2R | GCATCCTACTCTGCTCCGGCGGGGCTGCTGC |
| **V139I**mut-1F | TTCACTTCAGAAACCAG**A**TTAAAATACGCCGCAGG | Val-139 → Ile |
| **V139I**mut-2R | CCTGCGGCGTATTTTAATCTGGTTTCTGAAGTGAA |
| **K191E**mut-1F | TTACCAAGGAAAAGAAGGAGGAGCTTCAGGACTGC | Lys-191 → Glu |
| **K191E**mut-2R | GCAGTCCTGAAGCTCCTCCTTCTTTTCCTTGGTAA |
| **V511I**mut-1F | GAGACCAGAGGGGAAACATACTCCTGTCTCCTGAA | Val-511 → Ile |
| **V511I**mut-2R | TTCAGGAGACAGGAGTATGTTTCCCCTCTGGTCTC |
| **M535L**mut-1F | TAACATATTCATCGTCCCTGATGTGGGAGATCAAT | Met-535 → Leu |
| **M535L**mut-2R | ATTGATCTCCCACATCAGGGACGATGAATATGTTA |
| **N559T**mut-1F | CATTAGAAATTGGGAAACTGTAAAGATTCAATGGT | Asn-559 → Thr |
| **N559T**mut-2R | ACCATTGAATCTTTACA**G**TTTCCCAATTTCTAATG |
| **M570I**mut-1F | TCCCAAGATCCTACAATTCTATACAATAAGATGGA | Met-570 → Ile |
| **M570I**mut-2R | TCCATCTTATTGTATAGAATTGTAGGATCTTGGGA |
| **Q591K**mut-1F | CTAAAGCTGCCAGAGGC**A**AATATAGTGGGTTCGTG | Gln-591 → Lys |
| **Q591K**mut-2R | CACGAACCCACTATATTTGCCTCTGGCAGCTTTAG |
| **I647V**mut-1F | GGGGTTCCGGAATGAGAGTAGTTGTGAGAGGCAAT | Ile-647 → Val |
| **I647V**mut-2R | ATTGCCTCTCACAACTACTCTCATTCCGGAACCCC |
| **M676V**mut-1F | AGGATGCAGGTGCATTGGTGGAAGACCCCGATGAG | Met-676 →Val |
| **M676V**mut-2R | CTCATCGGGGTCTTCCACCAATGCACCTGCATCCT |
| **D701N**mut-1F | CTGATTCTGGGCAAAGAAAACAAAAGATATGGGCCAG | Asp-701 → Asn |
| **D701N**mut-2R | CTGGCCCATATCTTTTGTTTTCTTTGCCCAGAATCAG |

* Underlined nucleotides encode the amino acids that the wild-type virus will change into.
